# The Design of the AZO Conductive Layer on Microchannel Plate

**DOI:** 10.1186/s11671-021-03515-0

**Published:** 2021-04-07

**Authors:** Yuman Wang, Shulin Liu, Baojun Yan, Ming Qi, Kaile Wen, Binting Zhang, Jianyu Gu, Wenjing Yao

**Affiliations:** 1 School of Physics, Nanjing University, Nanjing, 210093 China; 2 State Key Laboratory of Particle Detection and Electronics, Institute of High Energy Physics, Chinese Academy of Sciences, Beijing, 100049 China; 3 University of Chinese Academy of Sciences, Beijing, 100049 China

**Keywords:** ALD-MCP, The AZO conductive layer, The working resistance, ZnO, Al_2_O_3_

## Abstract

When the resistivity of the AZO conductive layer is within the MCP resistance requirement, the interval of the Zn content is very narrow (70–73%) and difficult to control. Aiming at the characteristics of the AZO conductive layer on the microchannel plate, an algorithm is designed to adjust the ratio of the conductive material ZnO and the high resistance material Al2O3. We put forward the concept of the working resistance of the MCP (i.e., the resistance during the electron avalanche in the microchannel). The working resistance of AZO-ALD-MCP (Al2O3/ZnO atomic layer deposition microchannel plate) was measured for the first time by the MCP resistance test system. In comparison with the conventional MCP, we found that the resistance of AZO-ALD-MCP in working state and non-working state is very different, and as the voltage increases, the working resistance significantly decreases. Therefore, we proposed a set of analytical methods for the conductive layer. We also proposed to adjust the ratio of the conductive material of the ALD-MCP conductive layer to the high-resistance material under the working resistance condition, and successfully prepared high-gain AZO-ALD-MCP. This design opens the way for finding better materials for the conductive layer of ALD-MCP to improve the performance of MCP.

## Introduction

Microchannel plate (MCP) is an electron multiplier composed of two-dimensional pore arrays by thin glass plate form integration, length of 0.5–5 mm, a 4–40 μm diameter and with a bias angle usually 5°–13° to the normal of the plate surface; the open area ratio of the plate is up to 60%, and the high length-to-diameter ratio in each pore is about 20:1 to 100:1 [1].

**Fig. 1:**
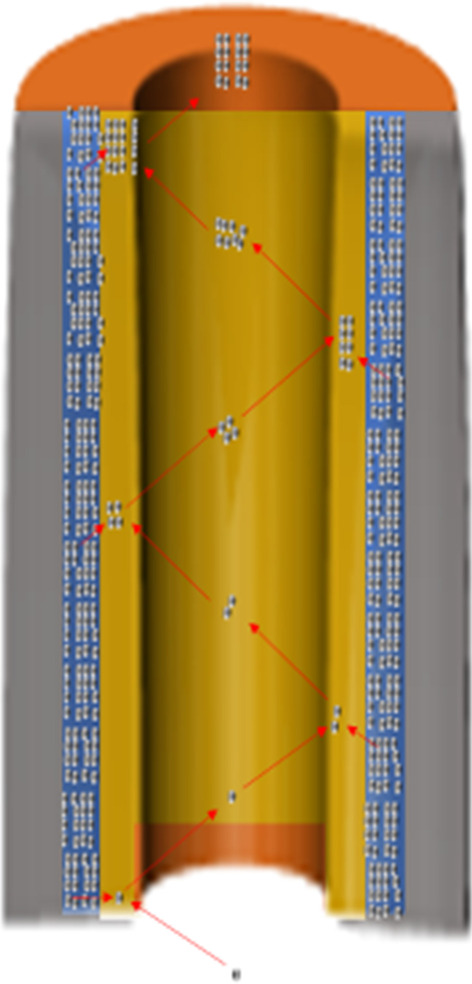
MCP working state diagram

As shown in Fig. 1, incident electrons entering the microchannel collide with the walls causing secondary electrons to be generated on the surface of the microchannel walls. Multi-collisions with the microchannel walls will lead to an increasing number of secondary electrons, resulting in an electron avalanche inside the microchannel and the emission of a cloud of electrons from the output of the microchannel. The secondary electron electrons will be further accelerated along the microchannel by a bias voltage. The MCP gain is 10^3^–10^4^ at a working voltage 700–900 V [2–9].

Each microchannel is as a detector and an electron multiplier. By having millions of microchannels working independently, MCP has the characteristics of high spatial resolution, high timing resolution and wide range of gain used to identify the photons, electrons, neutrons and ions. MCP can integrate into various kinds of instruments, including photoelectric detector, photomultiplier tubes (PMTs), ultraviolet spectrometer, cathode ray tube, scanning electron microscope, field emission displays, residual gas analyzer, medical imaging, time-of-flight mass spectrometry, night-vision goggles, etc. [1, 4, 7–9]. The hydrogen firing of the traditional process makes the microchannel suitable conductivity and secondary electron emission coefficient.

The usual process of hydrogen firing in the preparation of a microchannel has a lot of shortcomings: first, the hydrogen firing process cannot independently adjust the conductive layer and the emission layer [10, 11]; second, the heavy metal elements (Pb, Bi) lead to environmental pollution in the lead glass smelting process; third, large areas of MCP will become warped due to the high temperature [8]; fourth, lead glass being used hydrogen reduction reaction contains K, Rb and other radioactive elements resulting in background noise [8]; last, hydrogen which residues in the pores become ions due to bias voltage and they will fly in the opposite direction of electron to destroy the cathode of instrument [8, 12].

Early scientists propose a solution to grow the conductive layer and the emission layer on the microchannel wall to replace the hydrogen firing process [3]. Many thin film deposition methods are unable to grow a uniform film in the microchannel with high length-to-diameter ratios. The Argonne national laboratory proposed to use atomic layer deposition (ALD) to grow the conductive layer and the emission layer on the MCP to achieve an intact and uniform film on the microchannel walls [4, 13]. Furthermore, ALD-MCP solves the aforementioned shortcomings. Many research institutions are aiming towards finding competitive materials which can improve the performance of MCP.

The Argonne National Laboratory selects AZO materials for the ALD-MCP conductive layer taking into consideration the MCP resistance requirements. If the resistance is too high, the conductive layer cannot replenish electrons to the emission layer in time and continuously, the MCP will have low gains or even fail to operate. On the other hand, if the resistance is too low, the MCP will overheat, eventually leading to a breakdown [4, 9, 14, 15]. Hence, the design of the conductive layer is of importance for an ALD-MCP.

**Fig. 2:**
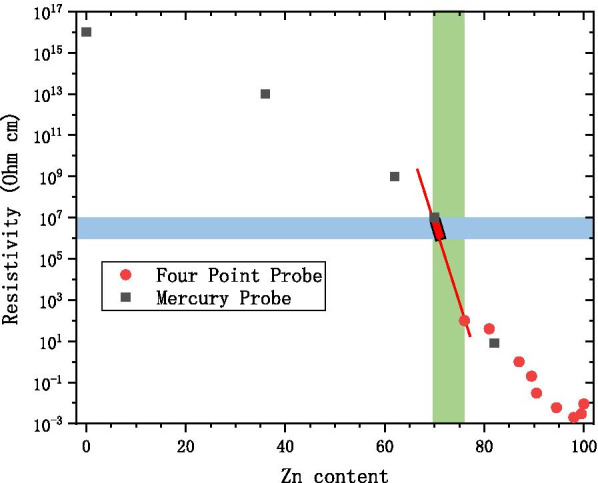
Zn content, Zn/(Zn + A)*100(%), blue area as the MCP resistance area, green area as AZO change area, the red areas as the need area to control

As shown in Fig. 2, when the resistivity of the AZO conductive layer is within the MCP resistance requirement, the allowed Zn content is in a very narrow range (70–73%) [16]. Hence, the MCP gain is unstable and the MCP can easily breakdown. Alternative conductive materials like W and Mo in place of Zn have been studied [3, 4, 17–19]. The chemical reaction of ${\text{WF}}_{6}$ (${\text{MoF}}_{6}$) and ${\text{H}}_{2} {\text{O}}$ is used to grow W (Mo) by ALD. However, using ${\text{WF}}_{6}$ or ${\text{MoF}}_{6}$ has two serious disadvantages: they are strongly corrosive and they contain impurities which can be difficult to remove during the production process. For these reasons, ALD-MCP with these materials is costly.

In our study, we find that reasonable designs with ZnO and ${\text{Al}}_{2} {\text{O}}_{3}$ can be realized for the MCP conductive layer, without the challenges faced if W or Mo is used, and is more competitive in price. Here, we name the ALD-MCP with an AZO conductive layer as AZO-ALD-MCP.

We propose an algorithm to adjust ratio of conductive material ZnO and high resistance material ${\text{Al}}_{2} {\text{O}}_{3}$ to obtain our desired AZO conductive layer characteristics.

We put forward the concept of the working resistance of the MCP (i.e., the resistance during the electron avalanche in the microchannel). We tested the working resistance of AZO-ALD-MCP and found two differences between AZO-ALD-MCPs and conventional MCPs. We observed that the working and non-working resistances of both AZO-ALD-MCPs and conventional MCPs are significantly different. Furthermore, the resistance of AZO-ALD-MLP is negatively correlated with the voltage. Our proposal (the reference to the working resistance) for adjusting the ratio of the conductive material and the high resistance material provides a guidance to help us to search for new materials to be used for the ALD-MCP conductive layer in improving the performance of the MCP in the future.

## Experimental and Methods

### Growing ZnO and ${\text{Al}}_{2} {\text{O}}_{3}$ Atomic Film

Atomic layer deposition (ALD) is a technology that alternates precursors and reactive gases to the surface of the substrate for physical or chemical adsorption or surface saturation reaction at a controlled rate. The material is deposited on the substrate in the form of a monoatomic film surface. ALD can produce a continuous film without pinholes, with excellent coverage, and can control the thickness and composition of the atomic film [1, 2, 4, 11, 13, 19, 20].

The following are the chemical reaction equations of using ALD to grow Al_2_O_3_:

$$\begin{aligned} & {\text{A}}:{\text{Substrate}} - {\text{OH}}^{*} + {\text{Al}}\left( {{\text{CH}}_{3} } \right)_{3} \\ & \quad \to {\text{Substrate}} - {\text{O}} - {\text{Al}}\left( {{\text{CH}}_{3} } \right)_{2}^{*} + {\text{CH}}_{4} \uparrow \\ & {\text{B}}:{\text{Substrate}} - {\text{O}} - {\text{Al}}\left( {{\text{CH}}_{3} } \right)_{2}^{*} + 2{\text{H}}_{2} {\text{O}} \\ & \quad \to {\text{Substrate}} - {\text{O}} - {\text{Al}}\left( {{\text{OH}}} \right)_{2}^{*} + 2{\text{CH}}_{4} \uparrow \\ & {\text{C}}:{\text{Al}} - {\text{OH}}^{*} + {\text{Al}}\left( {{\text{CH}}_{3} } \right)_{3} \\ { } & \quad \to {\text{Al}} - {\text{O}} - {\text{Al}}\left( {{\text{CH}}_{3} } \right)_{2}^{*} + {\text{CH}}_{4} \uparrow \\ & {\text{D}}:{\text{Al}} - {\text{CH}}_{3}^{*} + {\text{H}}_{2} {\text{O}} \to {\text{Al}} - {\text{OH}}^{*} + 2{\text{CH}}_{4} \uparrow \\ \end{aligned}$$

**Fig. 3:**
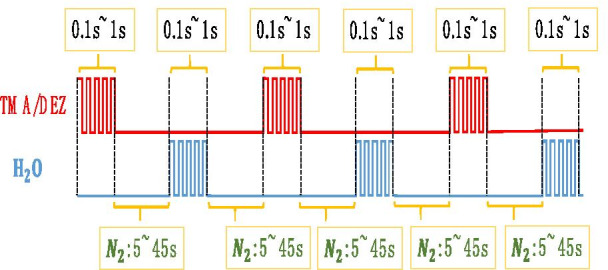
Growing Al_2_O_3_ and ZnO diagram

The temperature of the reaction is 60–150 °C. As shown in Fig. 3, the time and the order of growing a layer of Al_2_O_3_ atom is:

$${\text{TMA}}/{\text{N}}_{2} /{\text{H}}_{2} {\text{O}}/{\text{N}}_{2} = 0.1\sim1{\text{s}}/5\sim45{\text{s}}/0.1\sim1{\text{s }}/5\sim45{\text{s}}$$.

The following are the chemical reaction equations for using ALD to grow ZnO:

$$\begin{aligned} & {\text{E}}:{\text{Substrate}} - {\text{OH}}^{*} + {\text{Zn}}\left( {{\text{CH}}_{2} {\text{CH}}_{3} } \right)_{2} \\ & \quad \to {\text{Substrate}} - {\text{O}} - {\text{ZnCH}}_{2} {\text{CH}}_{3}^{*} + {\text{CH}}_{3} {\text{CH}}_{3} \uparrow \\ & {\text{F}}:{\text{Substrate}} - {\text{O}} - {\text{ZnCH}}_{2} {\text{CH}}_{3}^{*} + {\text{H}}_{2} {\text{O}} \\ & \quad \to {\text{Substrate}} - {\text{O}} - {\text{ZnOH}}^{*} + {\text{CH}}_{3} {\text{CH}}_{3} \uparrow \\ & {\text{G}}:{\text{Zn}} - {\text{OH}}^{*} + {\text{Zn}}\left( {{\text{CH}}_{2} {\text{CH}}_{3} } \right)_{2} \\ & \quad \to {\text{Zn}} - {\text{O}} - {\text{ZnCH}}_{2} {\text{CH}}_{3}^{*} + {\text{CH}}_{3} {\text{CH}}_{3} \uparrow \\ & {\text{H}}:{\text{Zn}} - {\text{CH}}_{2} {\text{CH}}_{3}^{*} + {\text{H}}_{2} {\text{O}} \to {\text{Zn}} - {\text{OH}}^{*} + {\text{CH}}_{3} {\text{CH}}_{3} \uparrow \\ \end{aligned}$$

The temperature of the reaction is 60–150 °C. As shown in Fig. 3, the time and the order of growing a layer of ZnO atom is:

$${\text{DEZ}}/{\text{N}}_{2} /{\text{H}}_{2} {\text{O}}/{\text{N}}_{2} = 0.1\sim1{\text{s}}/5\sim45{\text{s}}/0.1\sim1{\text{s }}/5\sim45{\text{s}}{.}$$

### Design of the AZO Conductive Layer

The thickness of the AZO usually ranges from 300 to 1000 atomic layers. We define a new mathematical operation rule to design the atomic layer orders of Al2O3 and ZnO in order to adjust the ratio of the conductive material ZnO and high resistance material Al2O3.

1 $$\left(\begin{array}{*{20}c} {{\text{mA}}} \\ {{\text{mB}}} \\ \vdots \\ \end{array} \right)= {\text{m}}\left(\begin{array}{*{20}c} {\text{A}} \\ {\text{B}} \\ \vdots \\ \end{array}\right)$$

2 $$\begin{aligned} & {\text{A}}\left(\begin{array}{*{20}c} {\text{a}} \\ {\text{b}} \\ \vdots \\ \end{array}\right) + {\text{B}}\left(\begin{array}{*{20}c} {\text{c}} \\ {\text{d}} \\ \vdots \\ \end{array}\right) + {\text{C}}\left(\begin{array}{*{20}c} {\text{e}} \\ {\text{f}} \\ \vdots \\ \end{array}\right) \ldots \\ & \quad = \left(\begin{array}{*{20}c} {\text{A}} \\ {\text{B}} \\ \vdots \\ \end{array}\right) \left[ \left(\begin{array}{*{20}c} {\text{a}} \\ {\text{b}} \\ \vdots \\ \end{array}\right) \left(\begin{array}{*{20}c} {\text{c}} \\ {\text{d}} \\ \vdots \\ \end{array}\right) \left(\begin{array}{*{20}c} {\text{e}} \\ {\text{f}} \\ \vdots \\ \end{array}\right) \ldots \right] = \left(\begin{array}{*{20}c} {{\text{Aa}} + {\text{Bc}} + {\text{Ce}} + \ldots } \\ {{\text{Ab}} + {\text{Bd}} + {\text{Cf}} + \ldots } \\ \vdots \\ \end{array}\right) \\ \end{aligned}$$

The mathematical operation was named WYM operation. WYM operation has two properties and a formula.

WYM property 1:

$$\begin{aligned} & \left( {\begin{array}{*{20}c} {\text{m}} \\ {\text{n}} \\ \end{array} } \right)\left[ {\left( {\begin{array}{*{20}c} {\text{a}} \\ {\text{b}} \\ \end{array} } \right)\left( {\begin{array}{*{20}c} {\text{c}} \\ {\text{d}} \\ \end{array} } \right)} \right]\left[ {\left( {\begin{array}{*{20}c} {\text{e}} \\ {\text{f}} \\ \end{array} } \right)\left( {\begin{array}{*{20}c} {\text{g}} \\ {\text{h}} \\ \end{array} } \right)} \right]\left[ {\left( {\begin{array}{*{20}c} {\text{i}} \\ {\text{j}} \\ \end{array} } \right)\left( {\begin{array}{*{20}c} {\text{k}} \\ {\text{l}} \\ \end{array} } \right)} \right] \ldots \\ & \quad = \left( {\begin{array}{*{20}c} {\text{m}} \\ {\text{n}} \\ \end{array} } \right)\left\{ {\left( {\begin{array}{*{20}c} {\text{a}} \\ {\text{b}} \\ \end{array} } \right)\left[ {\left( {\begin{array}{*{20}c} {\text{e}} \\ {\text{f}} \\ \end{array} } \right)\left( {\begin{array}{*{20}c} {\text{g}} \\ {\text{h}} \\ \end{array} } \right)} \right],\left( {\begin{array}{*{20}c} {\text{c}} \\ {\text{d}} \\ \end{array} } \right)\left[ {\left( {\begin{array}{*{20}c} {\text{e}} \\ {\text{f}} \\ \end{array} } \right)\left( {\begin{array}{*{20}c} {\text{g}} \\ {\text{h}} \\ \end{array} } \right)} \right]} \right\}\left[ {\left( {\begin{array}{*{20}c} {\text{i}} \\ {\text{j}} \\ \end{array} } \right)\left( {\begin{array}{*{20}c} {\text{k}} \\ {\text{l}} \\ \end{array} } \right)} \right] \ldots \\ & \quad = \left( {\begin{array}{*{20}c} {\text{m}} \\ {\text{n}} \\ \end{array} } \right)\left[ {\left( {\begin{array}{*{20}c} {\text{a}} \\ {\text{b}} \\ \end{array} } \right)\left( {\begin{array}{*{20}c} {\text{c}} \\ {\text{d}} \\ \end{array} } \right)} \right]\left\{ {\left( {\begin{array}{*{20}c} {\text{e}} \\ {\text{f}} \\ \end{array} } \right)\left[ {\left( {\begin{array}{*{20}c} {\text{i}} \\ {\text{j}} \\ \end{array} } \right)\left( {\begin{array}{*{20}c} {\text{k}} \\ {\text{l}} \\ \end{array} } \right)} \right],\left( {\begin{array}{*{20}c} {\text{g}} \\ {\text{h}} \\ \end{array} } \right)\left[ {\left( {\begin{array}{*{20}c} {\text{i}} \\ {\text{j}} \\ \end{array} } \right)\left( {\begin{array}{*{20}c} {\text{k}} \\ {\text{l}} \\ \end{array} } \right)} \right]} \right\} \ldots \\ \end{aligned}$$

WYM property 2:

$$\begin{aligned} & {\text{A}}\left( {\begin{array}{*{20}c} {\text{m}} \\ {\text{n}} \\ \end{array} } \right)\left[ {\left( {\begin{array}{*{20}c} {\text{a}} \\ {\text{b}} \\ \end{array} } \right)\left( {\begin{array}{*{20}c} {\text{c}} \\ {\text{d}} \\ \end{array} } \right)} \right]\left[ {\left( {\begin{array}{*{20}c} {\text{e}} \\ {\text{f}} \\ \end{array} } \right)\left( {\begin{array}{*{20}c} {\text{g}} \\ {\text{h}} \\ \end{array} } \right)} \right] \ldots \\ & \quad = \left( {\begin{array}{*{20}c} {{\text{Am}}} \\ {{\text{An}}} \\ \end{array} } \right)\left[ {\left( {\begin{array}{*{20}c} {\text{a}} \\ {\text{b}} \\ \end{array} } \right)\left( {\begin{array}{*{20}c} {\text{c}} \\ {\text{d}} \\ \end{array} } \right)} \right]\left[ {\left( {\begin{array}{*{20}c} {\text{e}} \\ {\text{f}} \\ \end{array} } \right)\left( {\begin{array}{*{20}c} {\text{g}} \\ {\text{h}} \\ \end{array} } \right)} \right] \ldots \\ & \quad = \left( {\begin{array}{*{20}c} {\text{m}} \\ {\text{n}} \\ \end{array} } \right)\left[ {{\text{A}}\left( {\begin{array}{*{20}c} {\text{a}} \\ {\text{b}} \\ \end{array} } \right),{\text{A}}\left( {\begin{array}{*{20}c} {\text{c}} \\ {\text{d}} \\ \end{array} } \right)} \right]\left[ {\left( {\begin{array}{*{20}c} {\text{e}} \\ {\text{f}} \\ \end{array} } \right)\left( {\begin{array}{*{20}c} {\text{g}} \\ {\text{h}} \\ \end{array} } \right)} \right] \ldots \\ & \quad = \left( {\begin{array}{*{20}c} {\text{m}} \\ {\text{n}} \\ \end{array} } \right)\left[ {\left( {\begin{array}{*{20}c} {\text{a}} \\ {\text{b}} \\ \end{array} } \right)\left( {\begin{array}{*{20}c} {\text{c}} \\ {\text{d}} \\ \end{array} } \right)} \right]\left[ {{\text{A}}\left( {\begin{array}{*{20}c} {\text{e}} \\ {\text{f}} \\ \end{array} } \right),{\text{A}}\left( {\begin{array}{*{20}c} {\text{g}} \\ {\text{h}} \\ \end{array} } \right)} \right] \ldots \\ \end{aligned}$$

WYM formula:

$$\begin{aligned} & \left(\begin{array}{*{20}c} {\text{a}} \\ {\text{b}} \\ \vdots \\ \end{array}\right) = \left(\begin{array}{*{20}c} {{\text{A}} + \frac{{\text{X}}}{{\text{Y}}}} \\ {\text{b}} \\ \vdots \\ \end{array}\right) \propto {\text{Y}}\left(\begin{array}{*{20}c} {{\text{A}} + \frac{{\text{X}}}{{\text{Y}}}} \\ {\text{b}} \\ \vdots \\ \end{array}\right) = \left( {\begin{array}{*{20}c} {{\text{Y}} - {\text{X}}} \\ {\text{X}} \\ \end{array} } \right)\left[ \left(\begin{array}{*{20}c} {\text{A}} \\ {\text{b}} \\ \vdots \\ \end{array}\right) \left(\begin{array}{*{20}c} {{\text{A}} + 1} \\ {\text{b}} \\ \vdots \\ \end{array} \right) \right] \\ & \left(\begin{array}{*{20}c} {\text{a}} \\ {\text{b}} \\ \vdots \\ \end{array}\right) = \left(\begin{array}{*{20}c} {\text{a}} \\ {{\text{B}} + \frac{{\text{X}}}{{\text{Y}}}} \\ \vdots \\ \end{array}\right) \propto {\text{Y}}\left(\begin{array}{*{20}c} {\text{a}} \\ {{\text{B}} + \frac{{\text{X}}}{{\text{Y}}}} \\ \vdots \\ \end{array}\right) = \left( {\begin{array}{*{20}c} {{\text{Y}} - {\text{X}}} \\ {\text{X}} \\ \end{array} } \right)\left[ \left(\begin{array}{*{20}c} {\text{a}} \\ {\text{B}} \\ \vdots \\ \end{array}\right) \left(\begin{array}{*{20}c} {\text{a}} \\ {{\text{B}} + 1} \\ \vdots \\ \end{array}\right) \right] \\ \end{aligned}$$

Note that, lowercase letters represent real numbers, while uppercase letters represent integers. In Examples 1 and 2, we show an execution of the operation.

*Example 1*

$$\left( {\begin{array}{*{20}c} {{\text{ZnO}}} \\ {{\text{Al}}_{2} {\text{O}}_{3} } \\ \end{array} } \right) = \left( {\begin{array}{*{20}c} {4 + \frac{1}{2}} \\ 1 \\ \end{array} } \right) \propto \left( {\begin{array}{*{20}c} 1 \\ 1 \\ \end{array} } \right)\left[ {\left( {\begin{array}{*{20}c} 4 \\ 1 \\ \end{array} } \right)\left( {\begin{array}{*{20}c} 5 \\ 1 \\ \end{array} } \right)} \right] = \left( {\begin{array}{*{20}c} 4 \\ 1 \\ \end{array} } \right) + \left( {\begin{array}{*{20}c} 5 \\ 1 \\ \end{array} } \right)$$

**Fig. 4:**
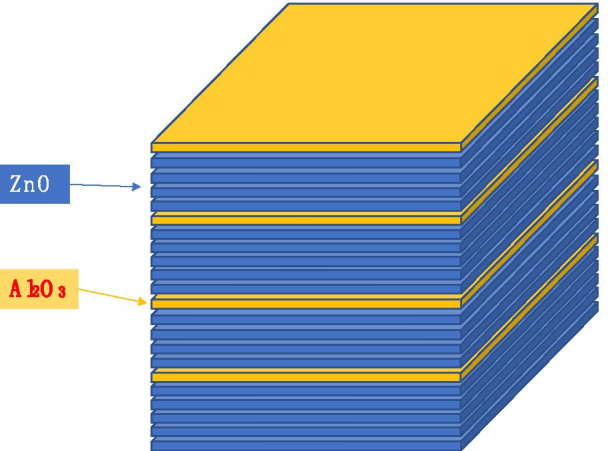
Schematic diagram of ZnO and Al_2_O_3_ growth sequence

The operation is interpreted as having two schemes: $$\left( {\begin{array}{*{20}c} 4 \\ 1 \\ \end{array} } \right)$$ and $$\left( {\begin{array}{*{20}c} 5 \\ 1 \\ \end{array} } \right)$$. For the first scheme, grow 4 times ZnO atomic layer and one Al2O3 atomic layer. For the second scheme, grow 5 times ZnO atomic layer and one Al2O3 atomic layer. If we repeat these two schemes twice, we will obtain the structure as shown in Fig. 4.

A more complicated usage of the operation rules is shown in Example 2, as follows:

$$\begin{aligned} & \left( {\begin{array}{*{20}c} {{\text{ZnO}}} \\ {{\text{Al}}_{2} {\text{O}}_{3} } \\ \end{array} } \right) = \left( {\begin{array}{*{20}c} {4.71} \\ 1 \\ \end{array} } \right) = \left( {\begin{array}{*{20}c} {4 + 0.71} \\ 1 \\ \end{array} } \right) \\ & \frac{2}{3} = 0.666 < 0.71 < \frac{3}{4} = 0.75 \\ & \left( {\begin{array}{*{20}c} E \\ F \\ \end{array} } \right)\left[ {\left( {\begin{array}{*{20}c} {4 + \frac{2}{3}} \\ 1 \\ \end{array} } \right)\left( {\begin{array}{*{20}c} {4 + \frac{3}{4}} \\ 1 \\ \end{array} } \right)} \right] \\ & \quad = E\left( {\begin{array}{*{20}c} {4 + \frac{2}{3}} \\ 1 \\ \end{array} } \right) + F\left( {\begin{array}{*{20}c} {4 + \frac{3}{4}} \\ 1 \\ \end{array} } \right) = \left( {\begin{array}{*{20}c} {4E + 4F + \frac{2}{3}E + \frac{3}{4}F} \\ {E + F} \\ \end{array} } \right) \\ & \quad = E + F\left( {\begin{array}{*{20}c} {4 + \frac{{\frac{2}{3}E + \frac{3}{4}F}}{E + F}} \\ 1 \\ \end{array} } \right) \propto \left( {\begin{array}{*{20}c} {4 + \frac{{\frac{2}{3}E + \frac{3}{4}F}}{E + F}} \\ 1 \\ \end{array} } \right) = \left( {\begin{array}{*{20}c} {4.71} \\ 1 \\ \end{array} } \right) \\ & \frac{{\frac{2}{3}E + \frac{3}{4}F}}{E + F} = 0.71 \Rightarrow {\text{E}} = 12,{\text{F}} = 13 \\ & \left( {\begin{array}{*{20}c} E \\ F \\ \end{array} } \right) = \left( {\begin{array}{*{20}c} {12} \\ {13} \\ \end{array} } \right) = 12\left( {\begin{array}{*{20}c} 1 \\ {1\frac{1}{12}} \\ \end{array} } \right) = \left( {\begin{array}{*{20}c} {11} \\ 1 \\ \end{array} } \right)\left[ {\left( {\begin{array}{*{20}c} 1 \\ 1 \\ \end{array} } \right)\left( {\begin{array}{*{20}c} 1 \\ 2 \\ \end{array} } \right)} \right] \\ & \left( {\begin{array}{*{20}c} E \\ F \\ \end{array} } \right)\left[ {\left( {\begin{array}{*{20}c} {4 + \frac{2}{3}} \\ 1 \\ \end{array} } \right),\left( {\begin{array}{*{20}c} {4 + \frac{3}{4}} \\ 1 \\ \end{array} } \right)} \right] \\ & \quad = \left( {\begin{array}{*{20}c} E \\ F \\ \end{array} } \right)\left[ {\left( {\begin{array}{*{20}c} 1 \\ 2 \\ \end{array} } \right)\left[ {\left( {\begin{array}{*{20}c} 4 \\ 1 \\ \end{array} } \right)\left( {\begin{array}{*{20}c} 5 \\ 1 \\ \end{array} } \right)} \right],\left( {\begin{array}{*{20}c} 1 \\ 3 \\ \end{array} } \right)\left[ {\left( {\begin{array}{*{20}c} 4 \\ 1 \\ \end{array} } \right)\left( {\begin{array}{*{20}c} 5 \\ 1 \\ \end{array} } \right)} \right]} \right] \\ & \left( {\begin{array}{*{20}c} {4.71} \\ 1 \\ \end{array} } \right) \propto \left( {\begin{array}{*{20}c} {12} \\ {13} \\ \end{array} } \right)\left[ {\left( {\begin{array}{*{20}c} 1 \\ 2 \\ \end{array} } \right)\left( {\begin{array}{*{20}c} 1 \\ 3 \\ \end{array} } \right)} \right]\left[ {\left( {\begin{array}{*{20}c} 4 \\ 1 \\ \end{array} } \right)\left( {\begin{array}{*{20}c} 5 \\ 1 \\ \end{array} } \right)} \right] = \left( {\begin{array}{*{20}c} {11} \\ 1 \\ \end{array} } \right)\left[ {\left( {\begin{array}{*{20}c} 1 \\ 1 \\ \end{array} } \right)\left( {\begin{array}{*{20}c} 1 \\ 2 \\ \end{array} } \right)} \right]\left[ {\left( {\begin{array}{*{20}c} 1 \\ 2 \\ \end{array} } \right)\left( {\begin{array}{*{20}c} 1 \\ 3 \\ \end{array} } \right)} \right]\left[ {\left( {\begin{array}{*{20}c} 4 \\ 1 \\ \end{array} } \right)\left( {\begin{array}{*{20}c} 5 \\ 1 \\ \end{array} } \right)} \right] \\ \end{aligned}$$

*Plan 1*: $$\left( {\begin{array}{*{20}c} {4.71} \\ 1 \\ \end{array} } \right) \propto 12\left[ {\left( {\begin{array}{*{20}c} 4 \\ 1 \\ \end{array} } \right) + 2\left( {\begin{array}{*{20}c} 5 \\ 1 \\ \end{array} } \right)} \right] + 13\left[ {\left( {\begin{array}{*{20}c} 4 \\ 1 \\ \end{array} } \right) + 3\left( {\begin{array}{*{20}c} 5 \\ 1 \\ \end{array} } \right)} \right]$$.

*Plan 2* $$\left( {\begin{array}{*{20}c} {4.71} \\ 1 \\ \end{array} } \right) \propto 11\left[ {\left[ {\left( {\begin{array}{*{20}c} 4 \\ 1 \\ \end{array} } \right) + 2\left( {\begin{array}{*{20}c} 5 \\ 1 \\ \end{array} } \right)} \right] + \left[ {\left( {\begin{array}{*{20}c} 4 \\ 1 \\ \end{array} } \right) + 3\left( {\begin{array}{*{20}c} 5 \\ 1 \\ \end{array} } \right)} \right]} \right] + \left[ {\left[ {\left( {\begin{array}{*{20}c} 4 \\ 1 \\ \end{array} } \right) + 2\left( {\begin{array}{*{20}c} 5 \\ 1 \\ \end{array} } \right)} \right] + 2\left[ {\left( {\begin{array}{*{20}c} 4 \\ 1 \\ \end{array} } \right) + 3\left( {\begin{array}{*{20}c} 5 \\ 1 \\ \end{array} } \right)} \right]} \right]$$.

In Example 2, the operation in Plan 1 can be interpreted as follows:

*Scheme 1* ALD grow 4 times ZnO atomic layer growth process and one ${\text{Al}}_{2} {\text{O}}_{3}$ atomic layer growth process; ALD grow 5 times ZnO atomic layer growth process and one ${\text{Al}}_{2} {\text{O}}_{3}$ atomic layer growth process, and repeat twice.

*Scheme 2* ALD grow 4 times ZnO atomic layer growth process and one ${\text{Al}}_{2} {\text{O}}_{3}$ atomic layer growth process; ALD grow 5 times ZnO atomic layer growth process and one ${\text{Al}}_{2} {\text{O}}_{3}$ atomic layer growth process, and repeat three times.

Repeat scheme 1 for 12 times, and scheme 2 for 13 times.

The interpretation of the operation in Plan 2 is along the same line as Plan 1.

### Microchannel Plate Resistance Test

**Fig. 5:**
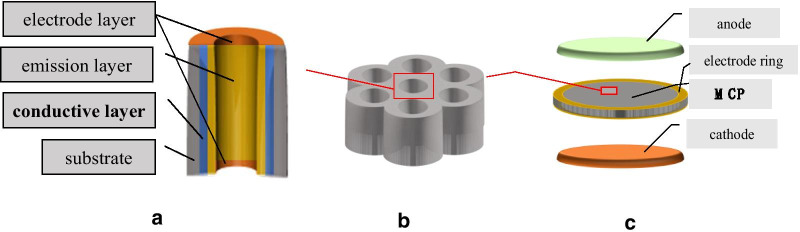
ALD–MCP resistance test schematic diagram

As shown in Fig. 5a, we use atomic layer deposition technology to grow the AZO conductive layer and the ${\text{Al}}_{2} {\text{O}}_{3}$ emission layer on microchannel walls of the two-dimensional pore arrays. And then we use thermal evaporation technology to grow the Ni–Cr electrode layer on the both sides of the MCP [2, 4] and put the electrode ring on the both sides of the MCP. Making preparations for the above, we directly test the ALD-MCP resistance. In this condition, we define the corresponding MCP resistance as the non-working resistance of the MCP. We use a Keithley model 6517B electrometer to measure the non-working resistance of the MCP in a 10^−3^–10^−5^ Pa vacuum [1, 4, 13].

As shown in Fig. 5c, we use an electron gun as the cathode and a phosphor screen as the anode. The electron gun provides incident electrons to the MCP, and the phosphor screen receives the electrons output by the MCP. In addition, when the MCP is under operation, the high-voltage phosphor screen will emit green light to detect the uniformity of the MCP [1, 21].

As shown in Fig. 1, we use an electron gun that provides a 100 pA as the input of the MCP to measure the current. Due to an increasing number of secondary electrons, there will be a condition where the emission layer loses a large amount of charges, and the conductive layer continuously provides a stream of charges to the emission layer. In this condition, we define the corresponding MCP resistance as the working resistance of the MCP. The vacuum environment of the working resistance is 10^−3^–10^−5^ Pa.

## Result and Discussion

**Fig. 6:**
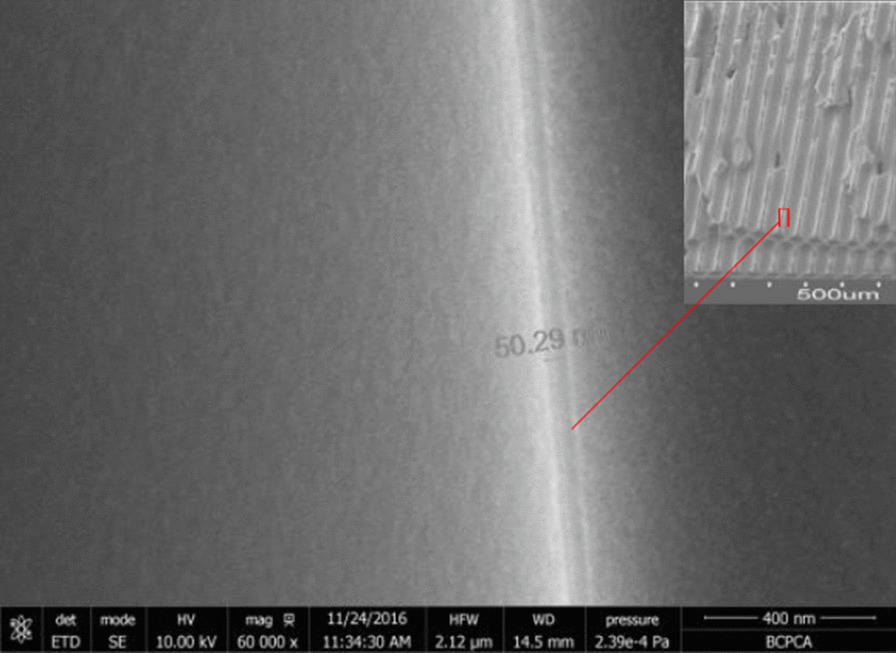
Cross-sectional SEM picture of the AZO-ALD-MCP

**Fig. 7:**
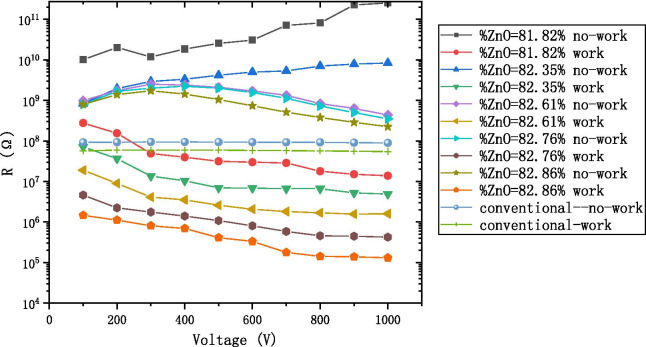
The working resistance and the non-work resistance with the voltage diagram at the AZO-ALD-MCP in the different ratio and conventional-MCP

The cross-sectional SEM picture of the AZO-ALD-MCP sample is shown in Fig. 6. We designed a series of AZO conductive layers as shown in Table 1 and their corresponding working and non-working resistances in Fig. 7. In the same figure, we also show the working and non-working resistances of a conventional MCP. In comparison with the non-working resistance of AZO-ALD-MCP, the working resistance of the AZO-ALD-MCP is significantly reduced. However, there is no significant difference between the working resistance and non-working resistance of a conventional MCP. As the voltage increases, the working resistance of AZO-ALD-MCP is significantly lower than that of a conventional MCP. Under the same voltage condition, the working and non-working resistances of the AZO-ALD-MCP are stable. We believe that there are two main reasons for the aforementioned characteristics.

According to formula [21],

$$R_{{{\text{MCP}}}} = R_{0} \exp \left[ { - \beta_{T} \left( {T_{{{\text{MCP}}}} - T_{0} } \right)} \right]$$

compared to lead glass, AZO is a material with a higher negative temperature coefficient (NTC), so the resistance will be lower at the same temperature and initial resistance. In the process of generating gain, AZO is bombarded by incident electrons at high voltage, thereby generating more electron–hole pairs, resulting in an increase in current.

**Table 1:** Detailed ALD experimental parameters for the AZO conductive layer

ZnO: ${\text{Al}}_{2} {\text{O}}_{3} = 4 + \frac{N}{N + 1}:1$	$$\left( {\begin{array}{*{20}c} {{\text{ZnO}}} \\ {{\text{Al}}_{2} {\text{O}}_{3} } \\ \end{array} } \right) = \left( {\begin{array}{*{20}c} 1 \\ N \\ \end{array} } \right)\left[ {\left( {\begin{array}{*{20}c} 4 \\ 1 \\ \end{array} } \right)\left( {\begin{array}{*{20}c} 5 \\ 1 \\ \end{array} } \right)} \right]$$	$$\left( {\begin{array}{*{20}c} {{\text{ZnO}}} \\ {{\text{Al}}_{2} {\text{O}}_{3} } \\ \end{array} } \right) = \left( {\begin{array}{*{20}c} 4 \\ 1 \\ \end{array} } \right) + N\left( {\begin{array}{*{20}c} 5 \\ 1 \\ \end{array} } \right)$$	$\% {\text{ZnO}} = \frac{{{\text{ZnO}}}}{{{\text{ZnO}} + {\text{Al}}_{2} {\text{O}}_{3} }}*100(\% )$
$4 + \frac{1}{2}:1$	$$\left( {\begin{array}{*{20}c} 1 \\ 1 \\ \end{array} } \right)\left[ {\left( {\begin{array}{*{20}c} 4 \\ 1 \\ \end{array} } \right)\left( {\begin{array}{*{20}c} 5 \\ 1 \\ \end{array} } \right)} \right]$$	$$\left( {\begin{array}{*{20}c} 4 \\ 1 \\ \end{array} } \right) + 1\left( {\begin{array}{*{20}c} 5 \\ 1 \\ \end{array} } \right)$$	81.82
$4 + \frac{2}{3}:1$	$$\left( {\begin{array}{*{20}c} 1 \\ 2 \\ \end{array} } \right)\left[ {\left( {\begin{array}{*{20}c} 4 \\ 1 \\ \end{array} } \right)\left( {\begin{array}{*{20}c} 5 \\ 1 \\ \end{array} } \right)} \right]$$	$$\left( {\begin{array}{*{20}c} 4 \\ 1 \\ \end{array} } \right) + 2\left( {\begin{array}{*{20}c} 5 \\ 1 \\ \end{array} } \right)$$	82.35
$4 + \frac{3}{4}:1$	$$\left( {\begin{array}{*{20}c} 1 \\ 3 \\ \end{array} } \right)\left[ {\left( {\begin{array}{*{20}c} 4 \\ 1 \\ \end{array} } \right)\left( {\begin{array}{*{20}c} 5 \\ 1 \\ \end{array} } \right)} \right]$$	$$\left( {\begin{array}{*{20}c} 4 \\ 1 \\ \end{array} } \right) + 3\left( {\begin{array}{*{20}c} 5 \\ 1 \\ \end{array} } \right)$$	82.61
$4 + \frac{4}{5}:1$	$$\left( {\begin{array}{*{20}c} 1 \\ 4 \\ \end{array} } \right)\left[ {\left( {\begin{array}{*{20}c} 4 \\ 1 \\ \end{array} } \right)\left( {\begin{array}{*{20}c} 5 \\ 1 \\ \end{array} } \right)} \right]$$	$$\left( {\begin{array}{*{20}c} 4 \\ 1 \\ \end{array} } \right) + 4\left( {\begin{array}{*{20}c} 5 \\ 1 \\ \end{array} } \right)$$	82.76
$4 + \frac{5}{6}:1$	$$\left( {\begin{array}{*{20}c} 1 \\ 5 \\ \end{array} } \right)\left[ {\left( {\begin{array}{*{20}c} 4 \\ 1 \\ \end{array} } \right)\left( {\begin{array}{*{20}c} 5 \\ 1 \\ \end{array} } \right)} \right]$$	$$\left( {\begin{array}{*{20}c} 4 \\ 1 \\ \end{array} } \right) + 5\left( {\begin{array}{*{20}c} 5 \\ 1 \\ \end{array} } \right)$$	82.86

We define the ratio of non-working resistance to working resistance to describe the stability of material resistance:

$$\kappa_{R} = \frac{{R_{n} }}{{R_{w} }}$$

**Fig. 8:**
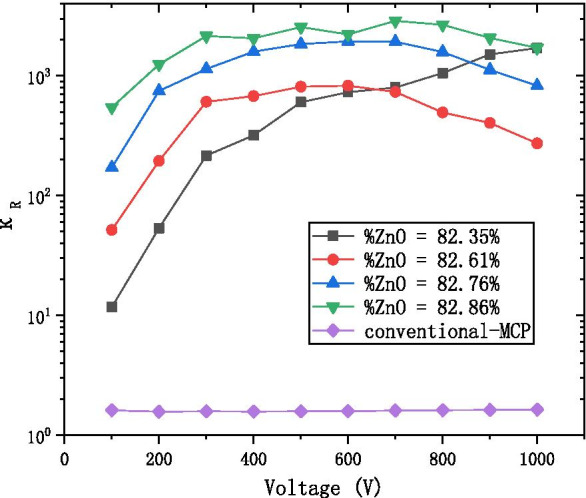
The *K*_*R*_ with the voltage diagram at the different ratio of AZO-ALD-MCP

Figure 8 shows that the $\kappa_{R}$ of AZO-ALD-MCP is about 10^2^–10^3^ times, and the $\kappa_{R}$ of conventional-MCP is about 2–3 times. This shows that the resistance change of AZO-ALD-MCP is more obvious; therefore, the old concept of non-working resistance as the definition for MCP resistance should be substituted with the working resistance instead.

**Fig. 9:**
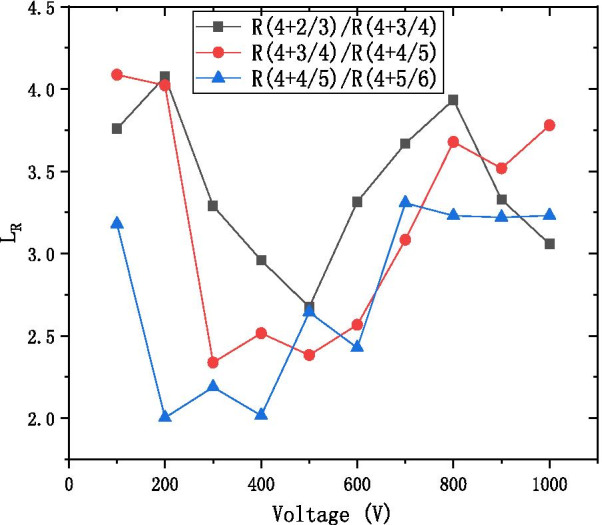
The resistance of the step length L_R_ with the voltage diagram at the different ratio of the working resistance of neighbor formula

Figure 9 shows the ratio $L_{R}$ of the resistance from “adjacent” material design with respect to the operating voltage. The ratio $L_{R}$ is defined to be:

$$L_{R} = \frac{{R\left( {4 + \frac{N - 1}{N}} \right)}}{{R\left( {4 + \frac{N}{N + 1}} \right)}}$$

where

$$\left( {\begin{array}{*{20}c} {{\text{ZnO}}} \\ {{\text{Al}}_{2} {\text{O}}_{3} } \\ \end{array} } \right) = \left( {\begin{array}{*{20}c} {4 + \frac{N - 1}{N}} \\ 1 \\ \end{array} } \right) = \left( {\begin{array}{*{20}c} 1 \\ {N - 1} \\ \end{array} } \right)\left[ {\left( {\begin{array}{*{20}c} 4 \\ 1 \\ \end{array} } \right)\left( {\begin{array}{*{20}c} 5 \\ 1 \\ \end{array} } \right)} \right]$$

and

$$\left( {\begin{array}{*{20}c} {{\text{ZnO}}} \\ {{\text{Al}}_{2} {\text{O}}_{3} } \\ \end{array} } \right) = \left( {\begin{array}{*{20}c} {4 + \frac{N}{N + 1}} \\ 1 \\ \end{array} } \right) = \left( {\begin{array}{*{20}c} 1 \\ N \\ \end{array} } \right)\left[ {\left( {\begin{array}{*{20}c} 4 \\ 1 \\ \end{array} } \right)\left( {\begin{array}{*{20}c} 5 \\ 1 \\ \end{array} } \right)} \right]$$

As can be observed from Fig. 9, the L_R_ value ranges from 2 to 4.5 to adjust ratio of conductive material ZnO and high resistance material ${\text{Al}}_{2} {\text{O}}_{3}$. And it proves the feasibility of WYM operation to design laminated materials.

**Fig. 10:**
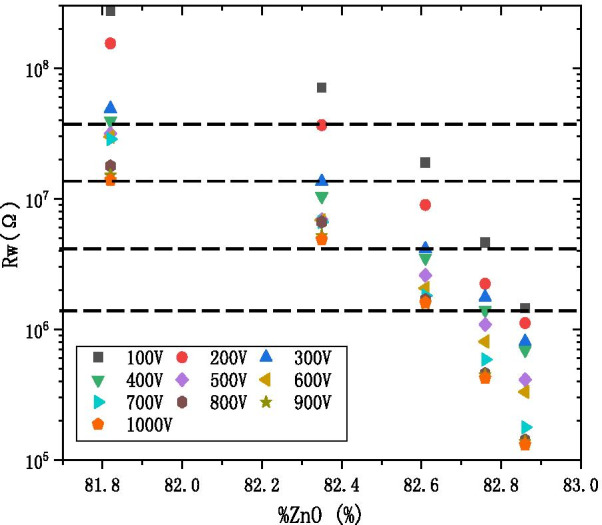
The working resistance with the percentage of ZnO cycles diagram at the different voltage

Figure 10 shows the working resistance with respect to the percentage of ZnO cycles (%ZnO), where %ZnO is defined to be:

$${\text{\% ZnO}} = \frac{{{\text{ZnO}}}}{{{\text{ZnO}} + {\text{Al}}_{2} {\text{O}}_{3} }}{*}100\left( {\text{\% }} \right)$$

under various voltage conditions, ranging from 100 to 1000 V. It decreases that the working resistance under the same voltage with the increase in the percentage of ZnO cycles. It can be the same that the working resistance under different the percentage of ZnO cycles and under the different condition of voltage. Therefore, the AZO-ALD-MCP of different formulations works under its specific voltage to meet the MCP resistance index.

We define the ratio of the resistance difference under the different condition of voltage and the voltage difference to describe the effect of the voltage on the resistance of MCP:

$$r = \left| {\frac{{R_{U} - R_{V} }}{U - V}} \right| = \left| {\frac{{R_{1000v} - R_{100v} }}{1000 - 100}} \right|$$

**Fig. 11:**
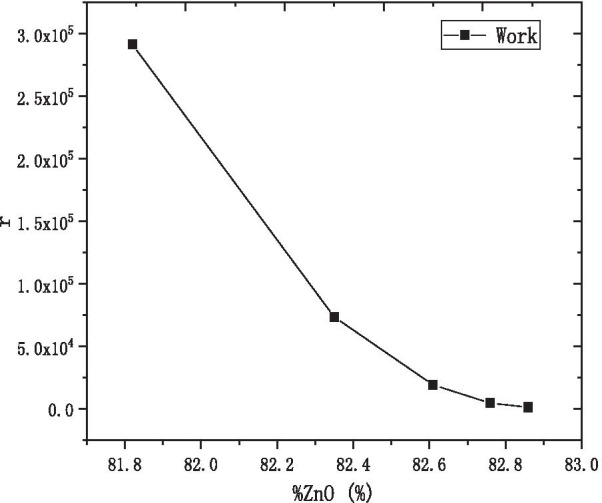
The r with the percentage of ZnO cycles diagram at the working state

Figure 11 shows that the effect of the voltage on the resistance of AZO-ALD-MCP decreased and gradually stabilized with the increase in the percentage of ZnO cycles. Therefore, the preparation of AZO-ALD-MCP should try to choose a formula with a large percentage of ZnO cycles.

**Fig. 12:**
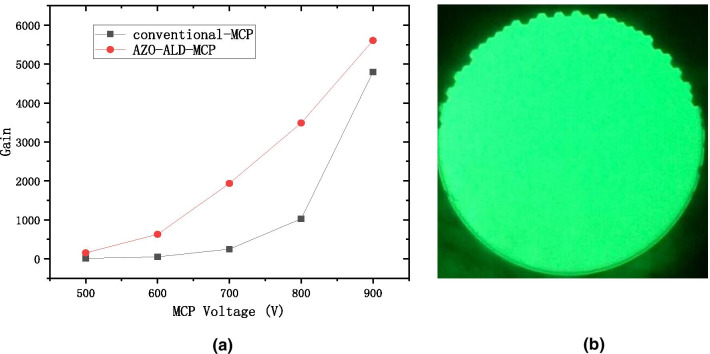
The gain with the voltage diagram at the AZO-ALD-MCP and conventional-MCP

Based on the above analysis, we have put forward the reference to the working resistance for the conductive layer of ALD-MCP. As shown in Fig. 5a, we design the AZO conductive layer of AZO-MCP by using the WYM operation and temperature adjustment based on the working resistance. We use atomic layer deposition technology to grow the ${\text{Al}}_{2} {\text{O}}_{3}$ emission layer on microchannel wall of the two-dimensional pore arrays [3, 11, 22]. In Fig. 12a, the gain from our AZO-ALD-MCP is compared to that of a conventional MCP under different voltages. As can be observed, our preparation method of the AZO-ALD-MCP provides a larger gain than that of a conventional MCP. Figure 12b shows the phosphor screen with uniform green light under high pressure, thus proving the uniformity of the material deposited on the wall of each microchannel and the uniformity of the AZO-ALD-MCP field of view.

## Conclusion

We defined the working and non-working resistance of the microchannel plate. Aiming at the required resistivity of the microchannel plate in the region with extremely narrow zinc content requirement (70–73%), an algorithm for growing the AZO conductive layer is proposed. Compared with the conventional MCP, we found a large difference between the working and non-working resistance and there is also a huge difference under different voltages. Therefore, we analyze the data by defining $\kappa_{R} ,L_{R} ,\% {\text{ZnO}},r$. MCP should try to choose a formula with a large percentage of ZnO cycles. We recommend using the working resistance as an ALD-MCP resistance indicator in industrial production. Building on our results as described in this work, our studies will help to find even better materials as the conductive layer for the ALD-MCP.
