# Correction to: The Design of the AZO Conductive Layer on Microchannel Plate

**DOI:** 10.1186/s11671-021-03556-5

**Published:** 2021-06-09

**Authors:** Yuman Wang, Shulin Liu, Baojun Yan, Ming Qi, Kaile Wen, Binting Zhang, Jianyu Gu, Wenjing Yao

**Affiliations:** 1 School of Physics, Nanjing University, Nanjing, 210093 China; 2 State Key Laboratory of Particle Detection and Electronics, Institute of High Energy Physics, Chinese Academy of Sciences, Beijing, 100049 China; 3 University of Chinese Academy of Sciences, Beijing, 100049 China

## Correction to: Nanoscale Res Lett (2021) 16:55 10.1186/s11671-021-03515-0

Following publication of the original article [1], the authors flagged that two of the numbers in Table 1 were incorrect.

The original article has now been updated with the corrected table. Please also find the corrected table in this article.

**Table 1:** Detailed ALD experimental parameters for the AZO conductive layer

ZnO: ${\text{Al}}_{2} {\text{O}}_{3} = 4 + \frac{N}{N + 1}:1$	$$\left( {\begin{array}{*{20}c} {{\text{ZnO}}} \\ {{\text{Al}}_{2} {\text{O}}_{3} } \\ \end{array} } \right) = \left( {\begin{array}{*{20}c} 1 \\ N \\ \end{array} } \right)\left[ {\left( {\begin{array}{*{20}c} 4 \\ 1 \\ \end{array} } \right)\left( {\begin{array}{*{20}c} 5 \\ 1 \\ \end{array} } \right)} \right]$$	$$\left( {\begin{array}{*{20}c} {{\text{ZnO}}} \\ {{\text{Al}}_{2} {\text{O}}_{3} } \\ \end{array} } \right) = \left( {\begin{array}{*{20}c} 4 \\ 1 \\ \end{array} } \right) + N\left( {\begin{array}{*{20}c} 5 \\ 1 \\ \end{array} } \right)$$	$\% {\text{ZnO}} = \frac{{{\text{ZnO}}}}{{{\text{ZnO}} + {\text{Al}}_{2} {\text{O}}_{3} }}*100(\% )$
$4 + \frac{1}{2}:1$	$$\left( {\begin{array}{*{20}c} 1 \\ 1 \\ \end{array} } \right)\left[ {\left( {\begin{array}{*{20}c} 4 \\ 1 \\ \end{array} } \right)\left( {\begin{array}{*{20}c} 5 \\ 1 \\ \end{array} } \right)} \right]$$	$$\left( {\begin{array}{*{20}c} 4 \\ 1 \\ \end{array} } \right) + 1\left( {\begin{array}{*{20}c} 5 \\ 1 \\ \end{array} } \right)$$	81.82
$4 + \frac{2}{3}:1$	$$\left( {\begin{array}{*{20}c} 1 \\ 2 \\ \end{array} } \right)\left[ {\left( {\begin{array}{*{20}c} 4 \\ 1 \\ \end{array} } \right)\left( {\begin{array}{*{20}c} 5 \\ 1 \\ \end{array} } \right)} \right]$$	$$\left( {\begin{array}{*{20}c} 4 \\ 1 \\ \end{array} } \right) + 2\left( {\begin{array}{*{20}c} 5 \\ 1 \\ \end{array} } \right)$$	82.35
$4 + \frac{3}{4}:1$	$$\left( {\begin{array}{*{20}c} 1 \\ 3 \\ \end{array} } \right)\left[ {\left( {\begin{array}{*{20}c} 4 \\ 1 \\ \end{array} } \right)\left( {\begin{array}{*{20}c} 5 \\ 1 \\ \end{array} } \right)} \right]$$	$$\left( {\begin{array}{*{20}c} 4 \\ 1 \\ \end{array} } \right) + 3\left( {\begin{array}{*{20}c} 5 \\ 1 \\ \end{array} } \right)$$	82.61
$4 + \frac{4}{5}:1$	$$\left( {\begin{array}{*{20}c} 1 \\ 4 \\ \end{array} } \right)\left[ {\left( {\begin{array}{*{20}c} 4 \\ 1 \\ \end{array} } \right)\left( {\begin{array}{*{20}c} 5 \\ 1 \\ \end{array} } \right)} \right]$$	$$\left( {\begin{array}{*{20}c} 4 \\ 1 \\ \end{array} } \right) + 4\left( {\begin{array}{*{20}c} 5 \\ 1 \\ \end{array} } \right)$$	82.76
$4 + \frac{5}{6}:1$	$$\left( {\begin{array}{*{20}c} 1 \\ 5 \\ \end{array} } \right)\left[ {\left( {\begin{array}{*{20}c} 4 \\ 1 \\ \end{array} } \right)\left( {\begin{array}{*{20}c} 5 \\ 1 \\ \end{array} } \right)} \right]$$	$$\left( {\begin{array}{*{20}c} 4 \\ 1 \\ \end{array} } \right) + 5\left( {\begin{array}{*{20}c} 5 \\ 1 \\ \end{array} } \right)$$	82.86

The authors apologize for any inconvenience caused.
